# Fluorodeoxyglucose positron emission tomography (FDG/PET) shows the effect of carbon-ion radiotherapy (CIRT); with pathologic confirmation

**DOI:** 10.1016/j.radcr.2023.02.007

**Published:** 2023-03-03

**Authors:** Jun Aoki, Yayoi Yamamoto, Ayumi Horikawa, Tsunehiro Doiuchi, Ayako Hino, Daichi Kojima, Hiroaki Kurihara, Kota Washimi, Hiroyuki Katoh, Toru Hiruma

**Affiliations:** a Department of Radiology and Interventional Radiology, Kanagawa Cancer Center, Yokohama, Kanagawa, Japan; b Department of Pathology, Kanagawa Cancer Center, Yokohama, Kanagawa, Japan; c Department of Radiation Oncology, Kanagawa Cancer Center, Yokohama, Kanagawa, Japan; d Department of Musculoskeletal Tumor Surgery, Kanagawa Cancer Center, Yokohama, Kanagawa, Japan

**Keywords:** FDG-PET, CT guided biopsy, Metastasis, Pleomorphic liposarcoma, Carbon-ion radiotherapy(CIRT)

## Abstract

Response evaluation of carbon-ion radiotherapy poses a diagnostic challenge. Due to its functional aspects, fluorodeoxyglucose positron emission tomography (FDG/PET) has a role in the diagnosis of photon radiation therapy. In addition, several studies suggested that FDG/PET may be useful to select the optimal site for performing a diagnostic biopsy. Here, we report a 73-year-old female in which FDG/PET was effective in determining the recurrence of liposarcoma and the therapeutic effect. Based on the results of FDG/PET, we could make a pathologic definitive diagnosis and selectively performing carbon-ion radiotherapy for active tumors.

## Introduction

Liposarcoma is one of the most common malignant soft tissue tumors. Liposarcoma tends to recur in various areas, and it is important how to determine recurrence and therapeutic effect in order to perform carbon-ion radiotherapy (CIRT) with an appropriate irradiation range.

Historically, surgery is one of the popular treatments for liposarcoma, but the number of cases treated by CIRT for recurrence of liposarcoma has also increased [1,2]. In this paper, we report a case in which fluorodeoxyglucose positron emission tomography (FDG/PET) was effective in determining the recurrence of liposarcoma and the therapeutic effect. Based on the results of FDG/PET, we could make a pathologic definitive diagnosis and selectively performing CIRT for active tumors.

## Case presentation

**Fig. 1:**
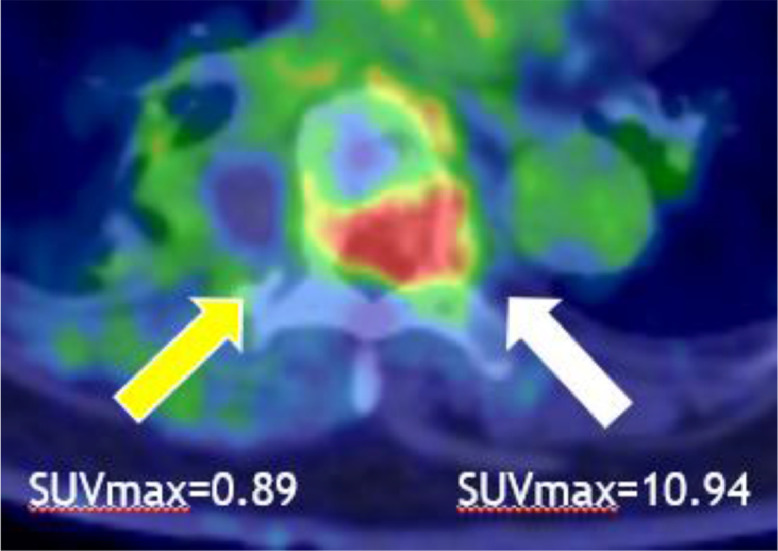
FDG/PET imaging findings. Axial FDG/PET image shows the mass of the Th7-Th9 not significant FDG uptake while the mass of 8th vertebra has significant FDG uptake with a maximum standardized uptake value (SUVmax) of 10.94.

**Fig. 2:**
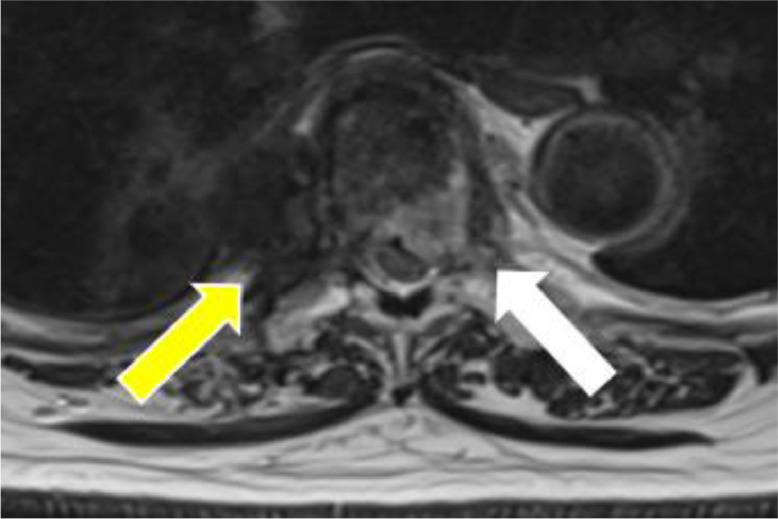
MRI imaging findings. Noncontrast T2-weighted MRI image shows a 35 × 17 × 25-mm mass in Th7-Th9 and a 20 × 24 × 19-mm mass in the Th8 vertebra.

The patient was a 73-year-old female who underwent wide excision for pleomorphic liposarcoma of her left humerus. About a year and a half after surgery, metastasis to her proximal eighth rib was found. CIRT (70.4 GyE/16FR) was performed for this metastasis. Subsequent follow-up FDG/PET showed no significant uptake in the post CIRT region, but significant uptake was observed in the eighth vertebra approximately 1 year after CIRT. The maximum standardized uptake value (SUVmax) of the eighth vertebra was 10.94 (Fig. 1). On the other hand, no significant accumulation was observed in the mass proximal to the eighth rib after CIRT. Nonenhanced MRI also revealed a 35 × 17 × 25 mm mass consistent with significant uptake in the eighth vertebral body. In addition, a Th7-Th9 compression fracture and a swelling change in the eighth rib were suspected (Fig. 2). From the above, it was presumed that the mass of the eighth vertebra, in which significant accumulation was observed, was metastasis from pleomorphic liposarcoma of the left humerus. A CT-guided percutaneous biopsy of the proximal eighth rib and eighth vertebra mass was performed to determine a more appropriate field for the upcoming second CIRT. As a result of pathologic diagnosis, the mass of the eighth vertebra was diagnosed as pleomorphic liposarcoma, which considered to be a metastasis from the primary lesion, pleomorphic liposarcoma of the left upper arm. On the other hand, the mass proximal to the eighth rib was necrotic tissue (Fig. 3a, Fig. 3b).

**Fig. 3a:**
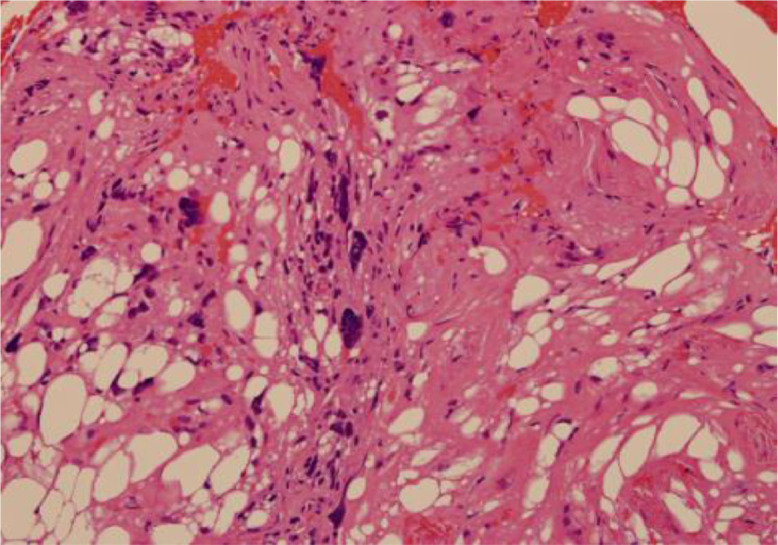
Pathologic findings of eighth vertebra (Hematoxylin and Eosin stain, original magnification is ×20). Large, atypical cells with vacuoles proliferate, accompanied by a large number of spindle cell proliferation and severe degeneration. This finding is consistent with liposarcoma.

**Fig. 3b:**
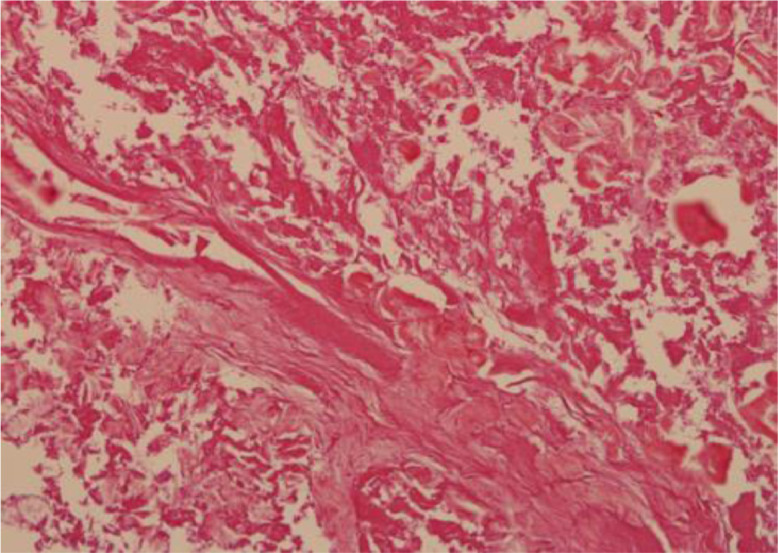
Pathologic findings of the proximal to the eighth rib (Hematoxylin and Eosin stain, original magnification is ×20). Numerous necrotic tissues are observed.

## Discussion

Response evaluation of CIRT poses a diagnostic challenge. CIRT may allow dose escalation to tumors compared to photon radiation therapy while reducing radiation dose to adjacent normal tissues for its favorable dose distribution [3]. Several promising safety and efficacy data are published, the imaging findings regarding CIRT response is not well discussed. Our report represents the efficacy of FDG/PET in determining the therapeutic effect and recurrence of liposarcoma.

As Nomia et al. [4] reported, the shrinkage of tumor is often slow, CT and MRI images provide small information for tumor activity. Due to its functional aspects, FDG/PET has a role in the diagnosis of photon radiation therapy [5]. In addition, several studies suggested that FDG/PET may be useful to select the optimal site for performing a diagnostic biopsy [5,6]. In our CIRT case, the proximal 8th rib, the initial irradiated cite, showed no significant FDG uptake. While, the 8th vertebra, suspected of metastasis, showed significant FDG/PET uptake. A CT-guided percutaneous needle biopsy of FDG-nonavid and avid lesion proved the accurate recurrent area.

CIRT is frequently considered for re-irradiation therapy due to their more favorable radiation dosimetry, which can often improve normal tissue sparing of organs at risk from additional radiation [7]. In our case, lung, vertebra, and thoracic cord, were the important adjacent tissues. The biopsy result was useful to exclude initial irradiated cite from second CIRT. FDG/PET played a critical role in establishing an accurate diagnosis and selective irradiated field.

## Conclusion

FDG-PET/CT was effective in determining the recurrence of liposarcoma and the therapeutic effect. FDG/PET was useful for making pathologic definitive diagnosis and performing CIRT for active tumors selectively.

## Patient consent

Written informed consent was obtained from the patient for their anonymized information to be published in this article.
